# Fetoscopic endoluminal tracheal occlusion (FETO) and bilateral congenital diaphragmatic hernia

**DOI:** 10.1515/crpm-2023-0010

**Published:** 2023-08-25

**Authors:** Adrita Khawash, Rania Kronfli, Anusha Arasu, Rashmi Gandhi, Kypros Nicolaides, Anne Greenough

**Affiliations:** Department of Women and Children’s Health, School of Life Course Sciences, Faculty of Life Sciences and Medicine, King’s College London, London, UK; Paediatric Surgery Department, King’s College Hospital NHS Foundation Trust, London, UK; Neonatal Intensive Care Centre, King’s College Hospital NHS Foundation Trust, London, UK; Department of Fetal Medicine, King’s College Hospital NHS Foundation Trust, London, UK

**Keywords:** bilateral congenital diaphragmatic hernia, FETO, duodenal atresia, patch repair

## Abstract

**Objectives:**

Bilateral congenital diaphragmatic hernias (CDH) occur in one to two percent of CDH patients. There is a lower survival due to the greater likelihood of lung hypoplasia and associated anomalies. We report an infant with bilateral CDH and duodenal atresia who was successfully treated by fetoscopic endoluminal tracheal occlusion (FETO).

**Case presentation:**

The fetus was diagnosed with CDH at 23 weeks of gestation. Her mother was referred to our tertiary centre as the observed to expected lung-to-head ratio (O/E LHR) at 26 weeks of gestation was only 17 %. The fetus was treated by FETO with an increase in the LHR. The mother had polyhydramnios and underwent amniotic fluid drainage at 26 and 31 weeks of gestation. She had preterm, premature rupture of the membranes at 31+3 weeks of gestation. The FETO balloon was punctured and the mother received corticosteroids. She underwent spontaneous labour at 35+6 weeks of gestation when the LHR was 55 %. At birth, the female infant was electively intubated and ventilated. After successful stabilisation, surgical intervention was undertaken on day six when the defects were identified as bilateral, type C posterolateral CDHs. Bilateral patch repair of the CDHs was undertaken using ‘domed’ Goretex patches. Type one duodenal atresia (DA) was identified and repaired with enterotomy and diamond duodenoduodenostomy. There was partial and then full abdominal closure on days 12 and 15 respectively. The infant is now four months of age and requires no respiratory support.

**Conclusions:**

FETO can improve prognosis in infants with bilateral CDH.

## Introduction

Congenital diaphragmatic hernia (CDH) is a major congenital defect which occurs in approximately one in 2000–3,000 live births. Rarely, in one to two percent of all CDH patients, it can be bilateral. Amongst 1833 patients reported in the CDH data base only 17 had bilateral CDHs [1]. In affected infants, survival may be less than 30 % due to the greater lung hypoplasia and the likelihood of associated anomalies [2]. We present an infant with severe bilateral CDH who was successfully treated by fetoscopic endoluminal tracheal occlusion (FETO) and had a favourable postoperative outcome.

### Case presentation

A 32 year old woman in her first pregnancy had an anomaly scan at 23 weeks of gestation, at which the fetus was diagnosed to have a right sided CDH. The pregnancy was also complicated by polyhydramnios. There was a normal fetal echocardiogram. The initial observed to expected (O/E) lung area to head circumference ratio (LHR) was 41 %, which reduced to 17 % at 26 weeks of gestation. The mother was then referred at 26 weeks of gestation to our Fetal Medicine Unit where a fetal endoluminal tracheal occlusion (FETO) procedure was performed. FETO was performed by placing a thin walled flexible Teflon cannula loaded with a custom designed pyramidal trocar into the amniotic cavity through the abdominal and uterine walls and directed towards the fetal mouth. The trocar was then withdrawn and fetoscopic instruments, including an endoscope, inserted. The endoscope was introduced into the fetal mouth, pharynx and epiglottis and advanced through the vocal cords to identify the carina. The catheter was positioned to deliver the balloon just above the carina. The procedure was performed under local anaesthetic. Post-FETO, the observed to expected LHR O/E increased to 90 %. At 26 and 31 weeks of gestation, amnio drainage was undertaken because of the polyhydramnios (1700 and 2,700 mLs respectively were removed). No chromosomal anomalies were detected on genetic testing undertaken by microarray analysis. At 31+3 weeks of gestation, there was preterm, premature rupture of the membranes. An *in-utero* transfer was undertaken to our tertiary perinatal medical and surgical unit. The FETO balloon was punctured *in-utero*. The mother was given a complete corticosteroid course (two doses of betamethasone 24 h apart). She subsequently went into spontaneous preterm labour at 35+6 weeks of gestation. The O/E LHR immediately prior to delivery was 55 %.

The female infant was born by a forceps assisted vaginal delivery. The infant’s birthweight was 2.37 kg and the Apgar scores were five at one minute and six at five minutes respectively. A nasogastric tube was inserted and the baby was electively intubated at two minutes of age. She was initially ventilated on conventional mechanical ventilation (CMV) with a fraction of inspired oxygen concentration of 0.60, but due to a rising pressure requirement due to hypercarbia she was transferred to high-frequency oscillatory ventilation (HFOV) until day three. In total, she was ventilated for 25 days and remained oxygen dependent up till discharge to her local hospital at two months of age.

A postnatal echocardiography showed a small intra-atrial communication and a PDA with bidirectional flow. Inhaled nitric oxide (iNO) was started on day one after birth due to a moderate pre-and post-ductal SpO2 difference of 5 %, but was able to be weaned after 48 h.

**Figure 1:**
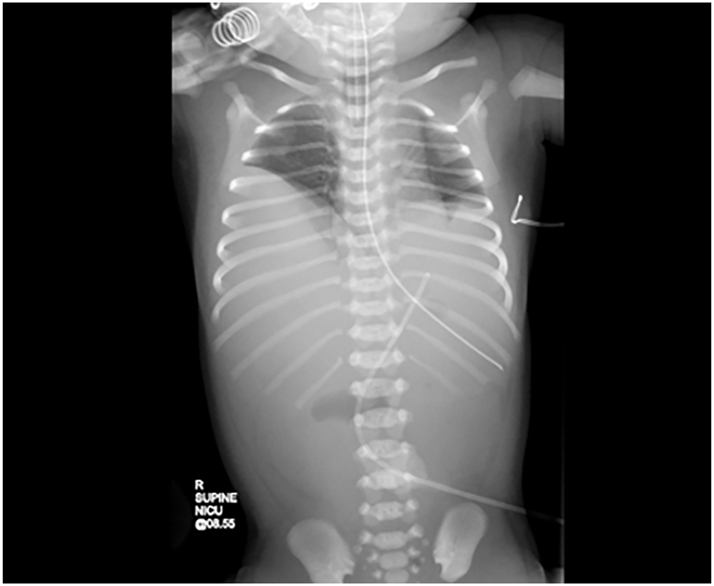
Chest radiograph demonstrating elevation of both hemidiaphragms; the small bilateral lungs are clear. There is a single dilated gas filled structure centrally in the mid-upper abdomen. The UVC is projected over the left upper quadrant of the abdomen.

**Figure 2:**
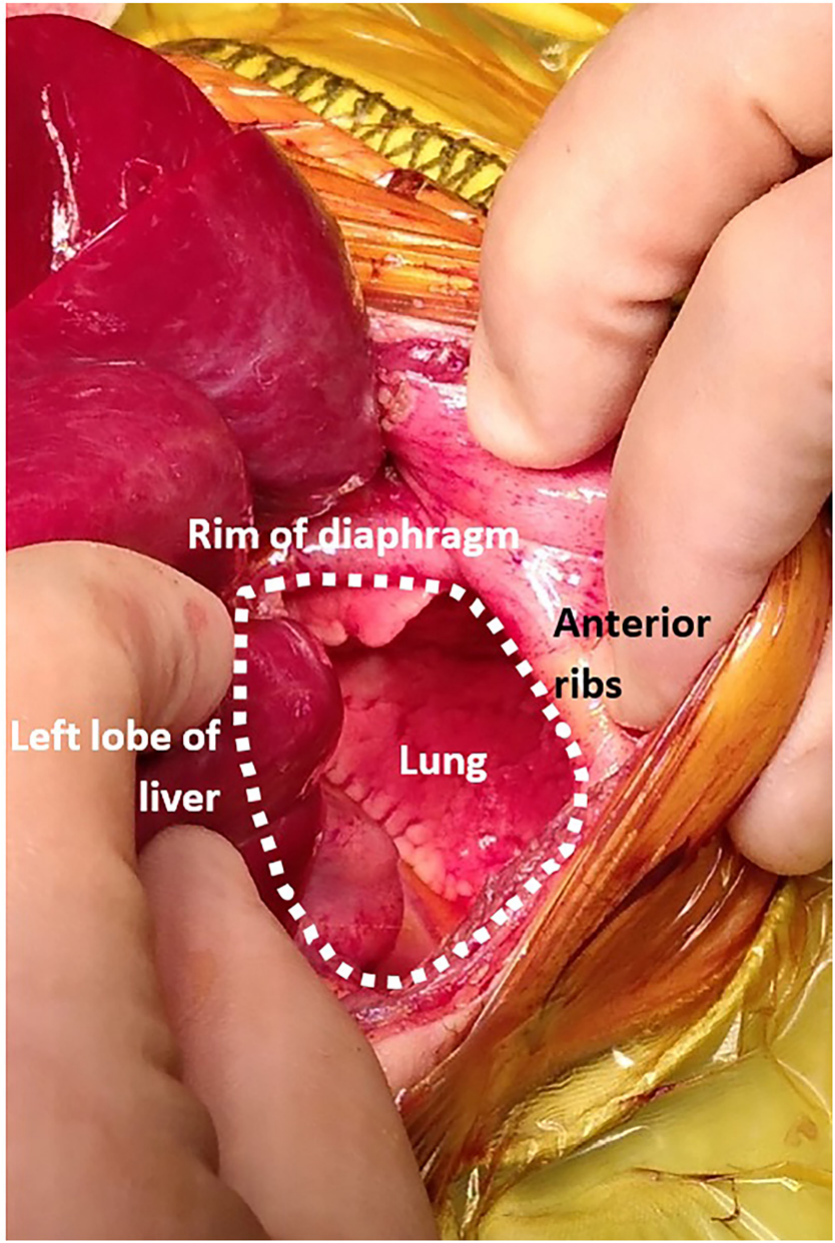
Operative picture demonstrating a large left sided defect (right sided the same) with a small anterior rim of diaphragm visible, reasonable sized lung and reduced left lobe of the liver.

The chest radiograph demonstrated elevation of both hemidiaphragms with clear small lung fields (Figure 1). The appearance on the chest ultrasound (USS) and CT Chest suggested a CDH and a contralateral eventration, but with the possibility that there could be bilateral CDH. The abdominal radiograph demonstrated a double bubble and no gas in the distal bowel loops and hence duodenal atresia (DA) was suspected. DA was confirmed by barium follow-through. Surgical repair was undertaken on day six. Bilateral, type C posterolateral CDHs were noted with herniation of the liver on the right and the spleen, stomach, colon, and left lobe of liver on the left (Figure 2). Surgical repair included bilateral patch repair of the CDHs using ‘domed’ Goretex patches, as primary repair was not possible due to the size of the defects. Type one duodenal atresia (DA), with no malrotation, was identified and repaired with enterotomy and diamond duodenoduodenostomy. In order to prevent abdominal compartment syndrome, the abdomen was kept open using a Goretex patch. The patch was reduced on day 12 and the abdomen was closed on day 15.

The infant no longer required respiratory support by two months of age. At an outpatient follow-up appointment at four months of age, she continued to require no respiratory support. The infant was gaining weight along the second per centile for age having a combination of breast feeding and bolus nasogastric feeds (Infatrini) via a pump.

## Discussion

We describe an infant successfully treated with FETO with bilateral type C Bochdalek hernias which we believe has not been previously reported. Bochdalek or posterolateral CDHs account for 80–90 % of all cases and are characterized by absent or deficient postero-lateral diaphragmatic muscle. Bilateral CDHs are more frequently associated with other major anomalies, for example absent pleura and pericardium. Molecular cytogenetic testing is therefore important as the prognosis is worse if multiple abnormalities are identified [3]. Our case had other congenital anomalies including duodenal atresia, but with no known syndromic association and normal genetic studies.

Review of 80 patients with bilateral CDHs reported to the CDH register highlighted a mortality rate of 74 %. Risk factors for adverse outcome were low Apgar scores, ECMO (probably as a surrogate for the severity of disease) and patch repair [4]. In our case, despite low Apgar scores and patch repair the infant survived.

In CDH patients, ultrasound assessment of LHR measurements and calculation of the observed to expected lung-to-head ratio (O/E LHR) has been used to predict postnatal survival and the need for ECMO in left-sided congenital diaphragmatic hernias [5]. In our case, the O/E LHR prior to FETO suggested severe hypoplasia [6]. The O/E LHR is determined from the contralateral lung and, as the fetus we describe had bilateral CDHs this likely resulted in the very low O/E LHR. There was, however, an increase in LHR ratio following the FETO procedure. Despite non-completion of the recommended duration of FETO due to puncture of FETO balloon following PPROM in anticipation of preterm labour, the observed to expected LHR was 55 % immediately prior to delivery at 35 weeks of gestation. FETO results in entrapment of lung fluid and this retained fluid activates stretch receptors and induces pulmonary proliferation and has been demonstrated to enhance lung maturation and reduce morbidity [6]. In the TOTAL trial, FETO was performed at 27–29 weeks of gestation in fetuses with isolated severe left CDH and resulted in a significant benefit over expectant care with respect to survival to discharge [6]. FETO, however, is associated with an increased risk of premature rupture of membranes and preterm birth as in this case. In 28 % of cases, the balloon needs to be removed prior to the recommended 34 weeks of gestation [6, 7]^.^

Initially postnatally, the infant was thought to have a CDH and a contralateral diaphragmatic eventration. Diaphragmatic eventration is due to defective diaphragmatic muscle development and often may coexist with a posterolateral CDH or they may be mistaken for each other. Both are associated with pulmonary hypoplasia and have been described to share a similar etiological origin. In this case, diagnostic imaging including chest ultrasound and CT Chest were not able to definitively diagnose the bilateral CDH. The problems of distinguishing bilateral CDH and one sided diaphragmatic eventration and a contralateral CDH have been previously reported [8]. In eventration, the elevated diaphragm can rise as high as the third intercostal space with similar radiological appearances and physiological consequences as CDH [8]. For similar reasons, it can be difficult to diagnose bilateral CDH prenatally, as in this case.

In conclusion, bilateral CDH is a very rare congenital complication with a very poor prognosis. In certain cases, fetoscopic endoluminal tracheal occlusion might improve prognosis.

## Take home message of the lessons learnt

Bilateral congenital diaphragmatic hernia is associated with a very poor prognosis.

It is often associated with other congenital anomalies and hence mothers with affected fetuses should be referred to a fetal medicine unit.

Fetoscopic endoluminal tracheal occlusion should be considered for such patients.
